# A Combination of Beta-Blockade and Calcium Channel Blockade Leading to Bradycardia, Renal Failure, Atrioventricular Blockade, Shock, and Hyperkalemia (BRASH) Syndrome: A Case Report

**DOI:** 10.7759/cureus.40176

**Published:** 2023-06-09

**Authors:** Kunj Patel, Varinder Singh, Andrew Bissonette

**Affiliations:** 1 Internal Medicine, Henry Ford Health System, Detroit, USA

**Keywords:** calcium channel blocker, beta blocker, hyperkalemia, atrioventricular blockade, renal failure, shock, bradycardia

## Abstract

The BRASH syndrome is a recently recognized syndrome and the acronym stands for bradycardia, renal failure, atrioventricular (AV) blockade, shock, and hyperkalemia. We discuss a case of a 56-year-old female with a history of heart failure who presented in a critical state following recent adjustments to her carvedilol dosage while she was simultaneously on verapamil. This combination of AV nodal-blocking agents induced bradycardia in the patient, leading to shock and renal hypoperfusion complicated by hyperkalemia that required the use of a temporary transvenous pacemaker before she made a full recovery. The case report highlights the fact that this combination of medications alone may have had a synergistic effect that led to BRASH in our patient.

## Introduction

The BRASH syndrome refers to a constellation of symptoms involving bradycardia, renal failure, atrioventricular (AV) blockade, shock, and hyperkalemia. It is a relatively new and underdiagnosed clinical finding. Its etiology is thought to be linked to a combination of hyperkalemia and AV nodal-blocking agents causing bradycardia. This leads to reduced cardiac output, which causes renal hypoperfusion and worsened hyperkalemia. This cycle progresses to multiorgan failure with renal failure, bradycardia, and shock [1]. In this report, we describe a case of BRASH syndrome in a patient with a history of heart failure with preserved ejection fraction (HFpEF) who had been recently admitted and treated for an acute exacerbation of HFpEF and had up-titration of her carvedilol while continuing with her verapamil, spironolactone, and lisinopril. This case illustrates the involvement of multiple AV nodal-blocking agents in addition to other medications that ultimately led to the patient’s presentation.

## Case presentation

A 56-year-old female with a past medical history of HFpEF, hypertension, and insulin-dependent type two diabetes mellitus was brought in by EMS with a complaint of feeling dizzy at home. She had a one-day history of generalized weakness and lethargy. Her family reported that she had subsequently become confused at home. She had been recently hospitalized one month ago with acute on chronic heart failure exacerbation and discharged home on verapamil 120 mg twice per day, carvedilol 12.5 mg twice per day, spironolactone 25 mg daily, furosemide 20 mg daily, and lisinopril 40 mg daily. She had also been seen by her cardiologist four days prior to the presentation. During that visit, her blood pressure had been 132/92 mmHg with a heart rate of 86 per minute. Her carvedilol dosage had been increased to 25 mg twice per day.

On presentation, her respiratory rate was 14 breaths per minute, her heart rate was 30 beats per minute, SpO_2_ was 92% on room air, the temperature was 32.9 °C, and her blood pressure was 70/55 mmHg. She initially had a Glasgow Coma Scale (GCS) score of 15 but became unresponsive soon after the presentation. The rest of her physical exam was unremarkable. Relevant positive findings on her initial laboratory results are presented in Table 1.

**Table 1:** Relevant lab values

Variable	Patient value (reference range)
Potassium	6.4 mmol/L (3.5-5.0)
Bicarbonate	20 mmol/L (21-35)
Blood urea nitrogen	58 mg/dL (10-25)
Creatinine	1.52 mg/dL (<1.16)
Glucose	549 mg/dL (60-140)
Lactic acid	7.3 mmol/L (<2.1)

**Figure 1:**
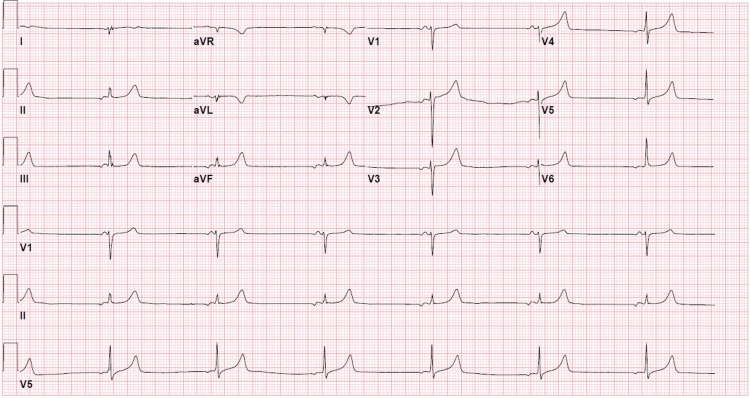
Initial ECG on presentation showing a heart rate of 40 beats per minute and peaked T-waves ECG: electrocardiogram

Electrocardiography (ECG) revealed a junctional escape rhythm with a heart rate of 40 beats per minute and peaked T-waves (Figure 1).

Her ECG at her office visit was reported to be in normal sinus rhythm with a heart rate of 86 beats per minute. There was increased suspicion of possible calcium channel blocker toxicity, and hence she was given IV glucagon. The patient was also given IV calcium gluconate, IV insulin, IV dextrose, and IV bicarbonate for hyperkalemia. She was given 2 mg of intravenous atropine for symptomatic bradycardia and put on a continuous epinephrine drip but did not show any improvement in her heart rate. Transcutaneous pacing was started, and the patient was intubated for airway protection due to her worsening GCS score. An arterial line and internal jugular Cordis catheter were placed. A transvenous pacemaker was placed and was set to a heart rate of 50 beats per minute with appropriate capture. The patient subsequently became hemodynamically stable, and epinephrine was weaned off. Her hyperkalemia improved with medical management, and she did not require urgent dialysis. Her lactic acid cleared with improvement in blood pressure after transvenous pacing was initiated. She maintained her heart rate above the set pacer and her transvenous pacemaker was removed two days later. Her mentation improved and she was successfully extubated.

## Discussion

The BRASH syndrome is a rarely described condition that involves a combination of bradycardia, renal failure, AV nodal blockade, shock, and hyperkalemia. It is linked to the association between hyperkalemia and AV nodal-blocking agents perpetuating bradycardia [1]. Bradycardia significantly impairs cardiac output, leading to decreased renal perfusion, and causing profound hyperkalemia. Thus, hyperkalemia and bradycardia contribute to systemic shock and can ultimately lead to death. The most common medications causing AV nodal blockade implicated in BRASH syndrome are calcium channel blockers and beta-blockers [2-3].

In our case, the calcium channel blocker involved was verapamil. Verapamil is a non-dihydropyridine calcium channel blocker that acts primarily on cardiac cells. It acts by inhibiting voltage-dependent L-type calcium channels [4-5]. By limiting intracellular calcium levels, verapamil reduces cellular conduction pathways as a type IV antidysrhythmic. This causes negative chronotropic and inotropic function in cardiac cells and vascular smooth muscle relaxation [5]. Toxic levels of verapamil lead to significantly decreased cardiac function and consequent acute kidney injury and hyperkalemia. It has also been implied that hyperkalemia can result from elevated calcium channel blocker levels, by altering transmembrane potassium channels directly [6].

Beta-blockers are also commonly implicated in this condition. Our patient was on carvedilol, a non-selective beta-blocker affecting both beta 1 and beta 2 receptors. By inhibiting beta-adrenergic receptors, carvedilol has negative chronotropic effects, which alone have been shown to precipitate BRASH syndrome, as well as vasodilating effects that can exacerbate hypotensive tissue perfusion [2-3]. That hyperkalemia can occur via extracellular potassium shift with the use of beta-blockers has been well-documented. However, there may be an additional calcium-related mechanism involved as well [7]. As a downstream signal transducer, calcium/calmodulin-dependent protein kinase II (CaMKII), which is a part of multiple pathways, has a potential role in beta-adrenergic regulation of cardiac function through cardiac dilation and hypertrophy [8-9]. However, there has been some evidence that it does not impact inotropy [9]. CaMKII tethers itself to L-type calcium channel blockers, the same pathway that verapamil acts upon [9]. Whether this transducer can have a summative effect in the setting of concurrent calcium channel blocker use is an area of uncertainty, and this phenomenon could have been involved in our patient’s presentation. 

Treatment for BRASH syndrome involves holding any medications potentially contributing to the pathophysiologic cycle, particularly antihypertensive medications, nephrotoxicity, and hyperkalemia. Medical management includes IV calcium for cardiac membrane stabilization, IV insulin and dextrose for hyperkalemia, and beta-agonist therapy with epinephrine or dopamine [1-2]. Renal replacement therapy and temporary pacemaker placement may be required in the most serious and refractory cases, which are rarely encountered [2].

## Conclusions

The BRASH syndrome is a potentially fatal phenomenon that may result from the synergistic effects of beta-blockers and non-dihydropyridine calcium channel blockers. A high index of suspicion for this potential pharmacologic interaction is required to rapidly diagnose and treat BRASH syndrome given the potential for clinical decompensation. This includes the use of dialysis or pacemakers in the most critical settings.

## References

[1] Farkas JD, Long B, Koyfman A, Menson K (2020). BRASH syndrome: bradycardia, renal failure, AV blockade, shock, and hyperkalemia. J Emerg Med.

[2] Majeed H, Khan U, Khan AM (2023). BRASH syndrome: a systematic review of reported cases. Curr Probl Cardiol.

[3] Shah P, Gozun M, Keitoku K, Kimura N, Yeo J, Czech T, Nishimura Y (2022). Clinical characteristics of BRASH syndrome: systematic scoping review. Eur J Intern Med.

[4] DeWitt CR, Waksman JC (2004). Pharmacology, pathophysiology and management of calcium channel blocker and beta-blocker toxicity. Toxicol Rev.

[5] Scholz H (1997). Pharmacological aspects of calcium channel blockers. Cardiovasc Drugs Ther.

[6] Ben Salem C, Badreddine A, Fathallah N, Slim R, Hmouda H (2014). Drug-induced hyperkalemia. Drug Saf.

[7] Martinez-Hernandez E, Kanaporis G, Blatter LA (2022). Mechanism of carvedilol induced action potential and calcium alternans. Channels (Austin).

[8] Grimm M, Brown JH (2010). Beta-adrenergic receptor signaling in the heart: role of CaMKII. J Mol Cell Cardiol.

[9] Schulman H, Anderson ME (2010). Ca/calmodulin-dependent protein kinase II in heart failure. Drug Discov Today Dis Mech.

